# Polymeric Materials in Energy Conversion and Storage, 2nd Edition

**DOI:** 10.3390/polym17222982

**Published:** 2025-11-10

**Authors:** Md Najib Alam, Vineet Kumar

**Affiliations:** School of Mechanical Engineering, Yeungnam University, 280, Daehak-ro, Gyeongsan 38541, Republic of Korea; mdnajib.alam3@gmail.com

## 1. Introduction

At present, the utilization of electronic devices is rapidly increasing due to the growing demand for a more sophisticated and convenient lifestyle. Consequently, the global demand for electrical energy is continuously rising. In general, mechanical and chemical energy sources serve as the two primary means of electrical energy generation. Although chemical energy can produce a substantial amount of electricity, it is often associated with environmental and safety concerns, prompting researchers to explore more sustainable and eco-friendly alternatives. Mechanical energy, which naturally exists in the form of wind or water flow, can be effectively converted into electrical energy through mechanical-to-electrical conversion systems such as wind turbines or hydroelectric generators. Likewise, renewable sources such as solar radiation and waste heat can be transformed into electricity using photovoltaic cells or thermoelectric generators, respectively, offering environmentally benign solutions. Moreover, efficient storage of electrical energy plays a crucial role in ensuring the continuous and stable operation of remote or portable electronic devices. Therefore, the development of advanced energy harvesting and storage systems has become an essential focus in modern energy research and technology.

The choice of materials plays a critical role in achieving efficient electrical energy conversion and storage. In this context, polymers have garnered significant attention due to their diverse range of physical and chemical properties. These materials exhibit remarkable versatility, with characteristics that vary from rigid to flexible, degradable to non-degradable, and stable under both low- and high-temperature conditions. Additionally, polymers offer tunable flow behavior, solvent swelling capacity, electrical conductivity or dielectric nature, chemical reactivity, and optical transparency. Their lightweight nature, ease of processing, and economic availability from both synthetic routes and natural sources further enhance their appeal. Owing to this unique combination of attributes, polymers have emerged as highly suitable candidates for energy generation and storage applications across a wide range of environmental and operational conditions.

Li et al. [1], in their review, discussed various polymers utilized in flexible energy storage devices. Polymer materials can serve multiple functions in these systems, acting as electrodes, electrolytes, separators, and packaging layers [1,2,3,4,5,6,7]. Similarly, Wang et al. [8] systematically described the role of polymers in self-powered energy conversion systems. Polymer-based energy harvesters have demonstrated broad applicability across diverse fields, including biomedical and electronic devices [8,9,10,11].

Several notable studies were reported in the previous Special Issue entitled “Polymeric Materials in Energy Conversion and Storage” [12]. Alam et al. [13,14] developed hybrid rubber composites using nano carbon black/MoS_2_ in silicone rubber and natural rubber/CNT–diatomaceous earth systems, achieving up to a 184% increase in fracture toughness and over a 100% enhancement in output voltage (3.2 mV), as well as a 484% improvement in toughness and a 25 mV output voltage for CNT-filled samples. Jeżowski et al. [15] fabricated a biopolymer membrane exhibiting a capacitance of 30 F/g and an output voltage of 1.6 V, while Guo et al. [16] synthesized redox-active polymers capable of generating up to 7.14 × 10^−4^ C of charge. Zappia et al. [17] employed silver nanoparticles to develop water-processable anodes, and Alshammari et al. [18] enhanced the electrical conductivity of PVA films by nearly an order of magnitude. Muñoz et al. [19] achieved 96.12% capacitance recovery in PVA/H_3_PO_4_-based supercapacitors, while Surisetty et al. [20] and Yuan et al. [21] investigated the aging and electrothermal behavior of polymers. Xu et al. [22] and Liu et al. [23] demonstrated ionogel actuators and PEDOT-based thermoelectric materials with a conductivity of 62.91 S/cm. Tameev et al. [24] reported that incorporating electron acceptor molecules such as PCBM into poly-TPD significantly enhanced charge carrier mobility, increasing electronic mobility from approximately 4.2 × 10^−6^ cm^2^/V·s for pure poly-TPD to 8.3 × 10^−6^ cm^2^/V·s for the poly-TPD:PCBM composite. Furthermore, Cho et al. [25] fabricated durable Zn–MnO_2_ batteries, and Hrostea et al. [26] along with Wei et al. [27] explored optoelectronic and light-actuated elastomer systems, demonstrating significant performance enhancements.

## 2. Overview of Published Articles

The continuous evolution of advanced materials for energy harvesting, conversion, and storage has accelerated the development of multifunctional devices and systems that merge mechanical robustness, structural adaptability, and high energy efficiency. Recent works [28,29,30,31,32,33,34,35,36] demonstrate substantial progress in these domains, spanning from structural design optimization in polymeric cellular architectures to hybrid and composite systems for triboelectric, piezoelectric, photovoltaic, and electrochemical applications. Collectively, these studies contribute to the growing body of knowledge focused on the design, processing, and multifunctional performance of next-generation materials.

Monkova et al. [28] investigated the mechanical behavior and energy absorption capacity of thermoplastic cellular structures based on triply periodic minimal surfaces (TPMS) such as Primitive, Diamond, and Gyroid architectures. Using Nylon CF12 fabricated via Fused Filament Fabrication (FFF), the study systematically evaluated the impact of volume fraction (30–55%) on bending properties, stiffness, yield strength, and effective modulus. The results highlighted the direct influence of cell topology and wall thickness on mechanical response and ductility indices, which are crucial for impact absorption and damping applications. Such findings underscore the promise of TPMS-based cellular structures in automotive, aerospace, and protective systems, where lightweight materials must exhibit high energy dissipation and durability.

Alam et al. [29] presented a highly efficient stretchable triboelectric nanogenerator (TENG) based on styrene–butadiene rubber (SBR) composites incorporating barium titanate (BT) and carbon nanotubes (CNTs). The study emphasizes the synergistic influence of dielectric and conductive fillers, particularly when BT is modified with stearic acid to enhance nanoscale dispersion. The resulting composites demonstrated superior power density (8.258 mW/m^3^) and charge efficiency (0.146 nC/N) under low compressive strain (2%), with stable performance under cyclic loading. Additionally, the TENG exhibited excellent handwriting recognition capabilities with high repeatability (<5% deviation), highlighting its potential in wearable electronics, low-pressure sensors, and human–machine interfaces. This work provides critical insights into achieving balance between mechanical flexibility and electrical performance in elastomer-based energy harvesters.

Li et al. [30] advanced the field of flexible piezoelectric sensors through the fabrication of ZnO nanorod (NR)-reinforced P(VDF-TrFE) nanofiber membranes. Employing electrospinning with a high-speed rotating drum, the researchers achieved uniform alignment and controlled nanorod incorporation. The optimal composition yielded a piezoelectric coefficient (d33) of −62.4 pC/N, nearly ten times higher than that of neat P(VDF-TrFE). The enhanced piezoelectric response facilitated effective bending and finger-tapping detection, demonstrating the potential of such nanocomposite membranes for wearable electronics, motion sensors, and self-powered devices. The study reinforces the efficacy of nanostructure–matrix interactions in tuning electromechanical coupling and offers a scalable route for the development of high-sensitivity piezoelectric materials.

Kim et al. [31] focused on self-powered perovskite-based photovoltaic photodiodes (PVPDs), integrating a poly(amic acid)-polyimide (PAA-PI) interfacial layer within MAPbI_3_ perovskite devices. The introduction of PAA-PI effectively reduced charge carrier recombination at the perovskite/PEDOT:PSS interface, yielding an improved power conversion efficiency (PCE) of 11.8%, compared to 10.4% for control samples. The devices exhibited significantly enhanced specific detectivity (7.82 × 10^10^ Jones) and rapid response times (61 µs rise; 18 µs decay). These improvements demonstrate the critical role of interfacial engineering in enhancing the optoelectronic properties of perovskite systems. Such design strategies pave the way for weak-light detection and high-sensitivity photodetectors, suitable for advanced imaging and sensing applications.

The study by Taouali et al. [32] employed density functional theory (DFT) and time-dependent DFT (TD-DFT) to explore novel donor–π–acceptor (D–π–A) organic compounds for solar cell applications. By modifying phenothiazine-based donors and introducing brominated thienyl-fused indanone-cyano (IC) acceptor groups, the researchers tailored six molecular systems (M1–M6) exhibiting HOMO–LUMO energy gaps between 2.14 and 2.30 eV and open-circuit voltages (Voc) exceeding 1.5 V. Enhanced dipole moments and strong charge transfer characteristics were achieved through halogen substitution, indicating potential for improved light absorption and charge separation. These computational insights serve as a roadmap for the rational design of high-efficiency organic photovoltaic materials, particularly those incorporating halogenated functionalities for better stability and tunability.

Feng et al. [33] provided an in-depth molecular-level understanding of proton exchange membranes (PEMs) by constructing semicrystalline and crystalline models of perfluoro sulfonic acid (PFSA) systems. Using molecular dynamics (MD) and energy-conserving dissipative particle dynamics (DPD) simulations, they revealed that crystalline regions exhibit 5–10 times higher proton transport efficiency and 1–3 times higher thermal conductivity compared to amorphous regions. The study introduced a proportionality coefficient dependent on temperature and hydration level to describe proton mobility, bridging computational predictions with experimental validation. The findings offer valuable insights into microstructure–transport relationships, facilitating the rational optimization of PEMs in fuel cells and hydrogen-based energy systems.

Fischer et al. [34] explored cellulose acetate butyrate (CAB) microspheres as a precursor for porous carbon materials, introducing an alternative to traditional cellulose acetate routes. CAB-based carbon microspheres exhibited high specific surface areas (567 m^2^/g) and tunable pore structures even without chemical activation. When employed as electrodes in symmetric supercapacitors (6 M KOH electrolyte), the activated carbons achieved energy densities of 12 Wh/kg at a power density of 0.9 kW/kg. These results highlight CAB-derived carbons as promising sustainable electrode materials with controllable morphology, bridging the gap between biopolymer chemistry and electrochemical energy storage.

Huang et al. [35] reviewed electrically conductive functional polymers (ECFPs)—notably conjugated and radical polymers—for applications in batteries, flexible electronics, and solid-state devices. The review detailed limitations such as low conductivity, dopant instability, and poor environmental resistance, and proposed strategies including molecular design, self-doping side chains, hydrophobic modifications, and 2D material composites. These approaches enhance electronic transport and mechanical resilience while promoting eco-friendly and renewable synthesis routes. The study underscores that tailoring the structure–function relationships in ECFPs is pivotal to achieving high-performance, sustainable energy storage materials.

Finally, Annu et al. [36] presented a comprehensive review of cobalt oxide composites with conducting polymers such as polypyrrole (PPy) and polyaniline (PANI) as electrode materials for supercapacitors, batteries, and hybrid “supercapatteries”. The synergy between cobalt oxide’s high theoretical capacitance and the polymers’ pseudocapacitive and conductive properties yields enhanced charge transport, ion diffusion, and cycling stability. Despite challenges in scalability and long-term durability, these hybrid systems show strong potential for next-generation electrochemical energy storage devices, bridging the functionality of batteries and supercapacitors.

## 3. Summary and Future Outlook

Collectively, these studies highlight a cross-disciplinary progression in the design, synthesis, and performance optimization of advanced materials across structural, electronic, and energy domains. From TPMS-based lightweight architectures to hybrid nanocomposites for triboelectric and piezoelectric energy harvesting and from molecularly engineered solar cells and photodiodes to biopolymer-derived carbons and conductive polymers for energy storage, a unifying trend becomes evident—namely, the convergence of multifunctionality, scalability, and sustainability. These collective innovations provide a strong foundation for the next generation of smart materials and devices capable of efficiently harvesting, storing, and utilizing energy while maintaining mechanical adaptability and environmental compatibility. Future research should focus on the development of functional polymers and filler systems with enhanced physical, chemical, and interfacial properties to further expand the potential of next-generation energy materials.
